# Zebrafish as a model to study autophagy and its role in skeletal development and disease

**DOI:** 10.1007/s00418-020-01917-2

**Published:** 2020-09-11

**Authors:** Joanna J. Moss, Chrissy L. Hammond, Jon D. Lane

**Affiliations:** 1 School of Biochemistry, Biomedical Sciences Building, University of Bristol, Bristol, UK; 2 School of Physiology, Pharmacology and Neuroscience, Biomedical Sciences Building, University of Bristol, Bristol, UK

**Keywords:** Autophagy, Zebrafish, Bone, Development, Tools

## Abstract

In the last twenty years, research using zebrafish as a model organism has increased immensely. With the many advantages that zebrafish offer such as high fecundity, optical transparency, ex vivo development, and genetic tractability, they are well suited to studying developmental processes and the effect of genetic mutations. More recently, zebrafish models have been used to study autophagy. This important protein degradation pathway is needed for cell and tissue homeostasis in a variety of contexts. Correspondingly, its dysregulation has been implicated in multiple diseases including skeletal disorders. In this review, we explore how zebrafish are being used to study autophagy in the context of skeletal development and disease, and the ways these areas are intersecting to help identify potential therapeutic targets for skeletal disorders.

## Introduction

### Autophagy

Autophagy is a catabolic process which enables the breakdown of cytosolic components into their basic biomolecular constituents by lysosomal degradation, so that they may be recycled for further use. It is an essential process required during cell differentiation and it contributes to the maintenance of cellular homeostasis where its primary function is to mobilise nutrients to sustain vital cellular functions during stress (Dikic and Elazar 2018). Since the first mechanistic descriptions of the autophagy process in 1967 by Christian de Duve (Deter and De Duve 1967), extensive research has been carried out to understand the autophagy pathways and their molecular control. Whilst these studies have established the importance of autophagy in cell differentiation and survival, they have also highlighted the vast number of housekeeping roles it plays, and the how its dysregulation contributes to the pathology of multiple diseases, including common skeletal disorders such as forms of arthritis and osteoporosis (Bouderlique et al. 2016; Cadwell and Debnath 2018; Jiang and Mizushima 2014; Levine and Kroemer 2019; Ochotny et al. 2013).

Autophagy can be divided into three main forms; chaperone-mediated autophagy (CMA), microautophagy and macroautophagy; each being delineated by the method of cargo delivery to the lysosome. This review will focus on macroautophagy (hereafter termed autophagy), as this considered to be the major form of autophagy and remains the most widely studied (Mizushima 2007). It involves the de novo formation of an intermediate organelle, the autophagosome, to deliver cargo to the lysosome for degradation. The autophagosome is a unique double-membrane structure which captures proteins, organelles, and other cellular debris before fusing with the lysosome to form a degradative autolysosome. This sequestration of cytosolic components can operate as a non-selective or selective process, with the former considered as a bulk, non-specific degradative process, whilst the latter requires specific receptor proteins to recognise and sequester target proteins, molecules, organelles, or invading pathogens.

### Mechanism of autophagy

The autophagy pathway is largely mediated by a family of highly conserved autophagy-related (ATG) proteins (Klionsky 2012; Wang et al. 2016), which were first discovered and characterised in budding yeast by Yoshinori Ohsumi and colleagues (Takeshige et al. 1992). To date, over 40 ATGs have been identified in yeast, most of which are conserved across higher eukaryotes (Kuma et al. 2017; Wei et al. 2018). Since their initial discovery, there has been an explosion of research focussed on delineating the fundamental mechanisms guiding the autophagic pathway. Broadly, this pathway can be split into three major steps: initiation and formation of the phagosome; phagosome elongation; and finally, lysosomal fusion. At each of these steps, dedicated ATG proteins and complexes are recruited and activated at distinct sites of phagophore assembly known as autophagosome initiation sites. As the nascent autophagosome is expanded, sealed, and trafficked to the lysosome, essential contributions are made by proteins co-opted from other cellular membrane trafficking pathways such as ESCRT complex proteins, tethers and SNAREs (Lamb et al. 2013).

**Fig. 1:**
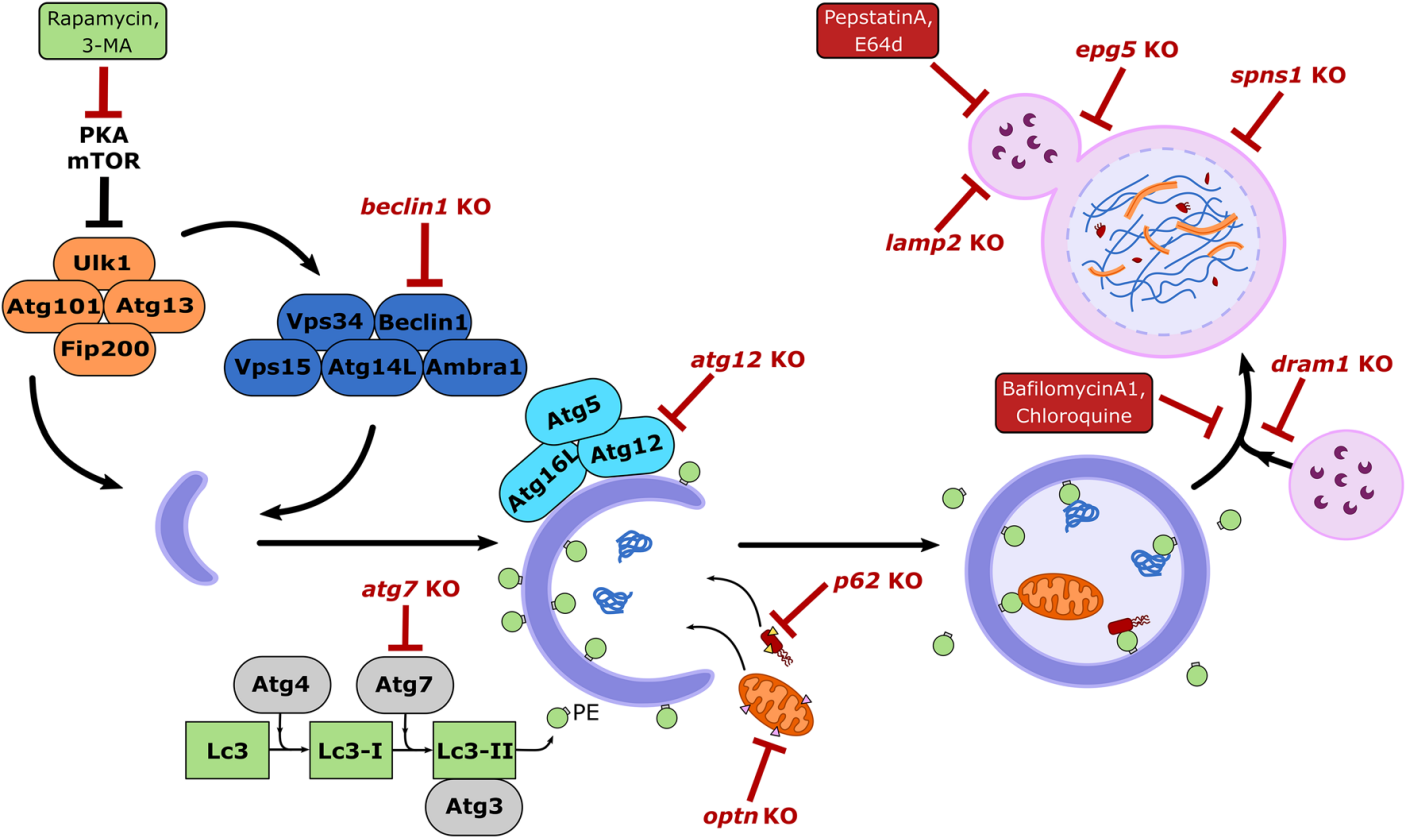
Overview of the core proteins involved in the autophagy pathway and its regulation in zebrafish, knockout (KO) zebrafish lines highlighted in red, and boxes show commonly used drugs which can activate (green) or inhibit (red) autophagy activity.

A comprehensive study of the full molecular machinery involved in the autophagy pathway is beyond the scope of this review (other in-depth reviews can be found here Boya et al. 2013; Dikic and Elazar 2018)). Instead, we will focus on the mammalian biology of core proteins thus far relevant to zebrafish autophagy models (Fig. 1). These include, the ULK1 complex, consisting of ULK1, ATG13, FIP200 and ATG101, and the phosphatidyl inositol 3-kinase complex I (PI3KC3), comprising VPS34, Beclin1 (BECN1), ATG14, AMBRA1 and p115, which are involved in autophagy initiation and phagophore formation. Next, the two conjugation systems: ATG5–ATG12-ATG16L and MAP1LC3–ATG3 help cooperatively recruit and conjugate MAP1LC3 to the lipid phosphatidylethanolamine (PE) present on the phagophore membrane to form lipidated MAP1LC3-II. Prior to this step, ATG4 and ATG7 are responsible for processing MAP1LC3 into MAP1LC3-I ready for its lipid conjugation. These steps are central for autophagy detection and analysis, as fluorescently labelled MAP1LC3 – which appears as discrete 0.5–1.0 µm puncta during autophagy— is the most commonly used marker for monitoring autophagy activity in cells and whole organisms. Finally, in the case of selective autophagy, receptor proteins such as p62/SQSTM-1, optineurin and NDP52 can specifically recognise and target polyubiquitinated cargo to the autophagosome.

Alongside these proteins, there are many other signalling pathways involved in initiating the autophagy response. Central to these are the mechanistic target of rapamycin complex 1 (mTORC1) pathway and the cAMP-dependent protein kinase A (PKA) pathway (Blommaart et al. 1995). It has become evident from a variety of studies in different organisms that the proper regulation of autophagy initiation is essential for maintaining cellular homeostasis. Indeed, many studies have implicated autophagy dysregulation in the development of multiple neurological, cardiovascular, metabolic, and more recently, skeletal diseases (Choi et al. 2013; Wang et al. 2016; Wirawan et al. 2012). Meanwhile, other studies have uncovered a diversity of other cellular functions, beyond protein degradation, which are mediated by the autophagy machinery (Cadwell and Debnath 2018; Levine and Kroemer 2019). These include processes such as cellular differentiation and proliferation, cell metabolism, ER stress mitigation, and non-cell autonomous nutrient mobilisation, all of which are essential to bone and cartilage cell development and survival (Carames et al. 2010; Lee et al. 2015).

By understanding the distinct functions performed by autophagy in specialised cells and the key factors involved in its regulation, we can better grasp the influence of autophagy during the development of skeletal diseases, and how the autophagic pathway can be manipulated for therapeutic benefit. This review will focus on how zebrafish can be used to study autophagy in the context of skeletal development and the tools and techniques currently available within this area of research.

## Autophagy and skeletal development

**Fig. 2:**
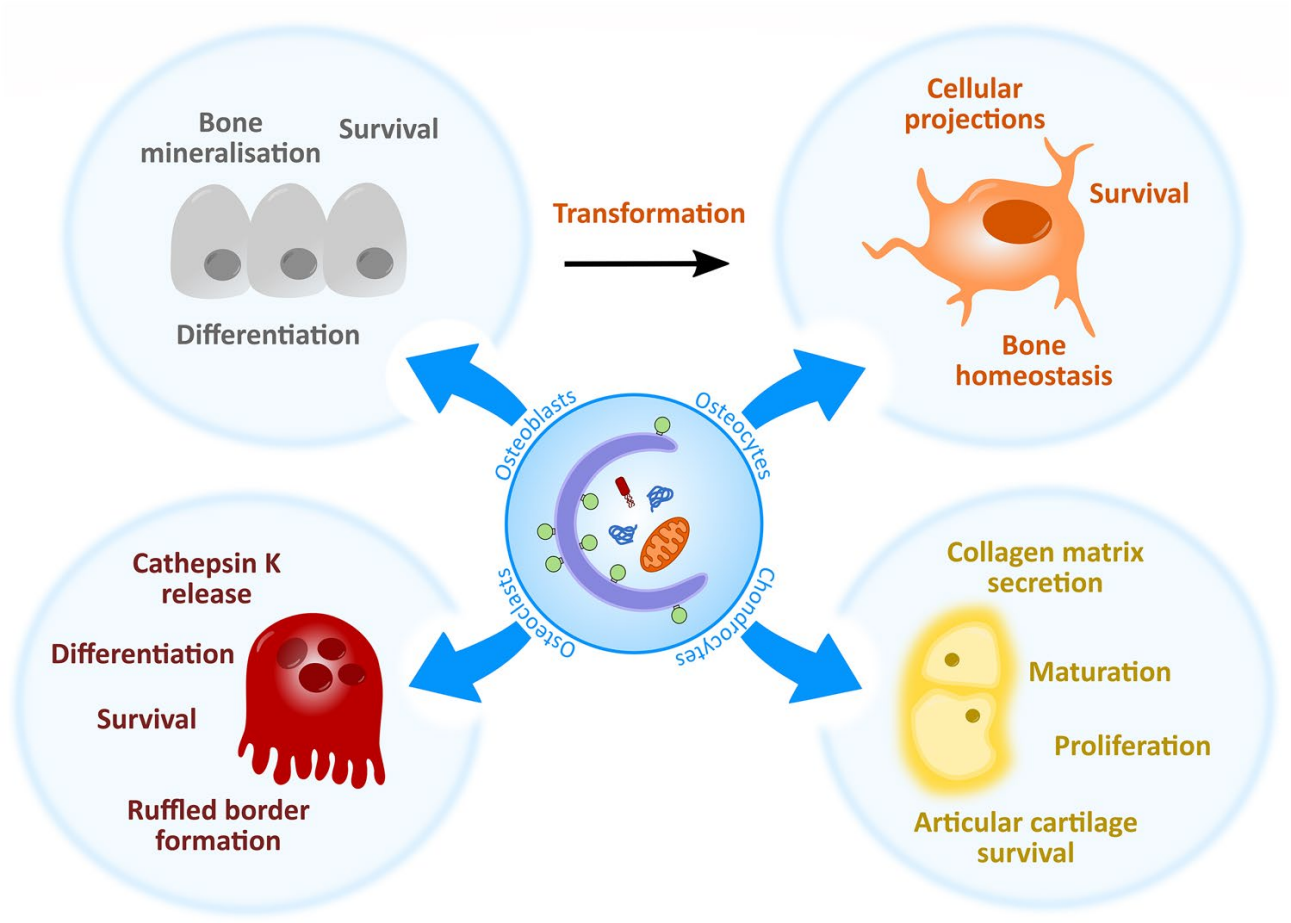
Overview of the roles autophagy plays in bone and cartilage cells, autophagy helps maintain the homeostasis, survival and function of osteoblasts, osteoclasts, osteocytes and chondrocytes

Multiple studies have implicated autophagy in the development and maintenance of the skeletal system. From the very early stages of development, autophagy plays essential roles in the differentiation, transformation and functional activity of key skeletal cells, such as osteoblasts (bone secreting cells), osteoclasts (bone absorbing cells), osteocytes (bone maintenance cells) and chondrocytes (cartilage secreting cells) (Fig. 2) (Aghajanian and Mohan 2018). Post-development, emerging evidence shows that once terminally differentiated, these cells require a constitutive level of basal autophagy to ensure proper functioning and survival in the hypoxic, nutrition-deficient and hypertonic environments they reside in (Mizushima and Levine 2010).

During embryonic development, the vertebrate skeleton, and its associated bone, cartilage, and connective tissues, are formed from mesenchymal stem cells (MSCs). These cells are originally derived from three distinct embryonic cell lineages which go on to establish different regions of the skeleton (Hall and Miyake 1992). To form the skeleton, cells from these lineages first migrate to the appropriate region within the embryo for skeletal formation, where they aggregate and proliferate to form mesenchymal condensations that subsequently differentiate into either osteoblasts or chondrocytes. Studies have highlighted vital roles for autophagy within this differentiation process and within the differentiation capabilities of MSCs. For example, MSCs have been shown to have high levels of basal autophagy (Oliver et al. 2012), whilst treatment of MSCs isolated from young mice with the class III PI3Kinase inhibitor 3-methyladenine (3-MA), which blocks autophagy induction, was shown to cause a reduction in the capacity of MSCs to differentiate into osteoblasts (Ma et al. 2018). Further to this, a study in primary human MSCs showed that during osteoblast differentiation from MSCs, these cells accumulate large numbers of autophagic vacuoles which are later broken down to provide energy (Nollet et al. 2014). The authors concluded that autophagy is required to help balance energy supply during the differentiation process and that it is therefore vital for MSC differentiation and function (Nuschke et al. 2014). An in vitro study using primary murine osteoblast cells also showed that blocking autophagy through the knockout of FIP200, an essential component of the mammalian ULK1 complex, inhibited osteoblast differentiation, further demonstrating the importance of autophagy during the establishment of the osteoblast population (Liu et al. 2013).

From osteoblast and chondrocyte cells, bone is formed via two different mechanisms. In intramembranous ossification, bone is secreted directly by osteoblasts which is how much of the craniofacial skeleton, vertebral column, and fins are formed in zebrafish (Bird and Mabee 2003). Conversely, in endochondral ossification, chondrocytes first form a cartilaginous skeletal template which is then gradually replaced by bone. For tetrapods, this is how long bones form, whilst in zebrafish the ceratohyal and hyperurals are formed this way (Mundlos and Olsen 1997). As the organism develops, bone is lengthened and modelled until the final skeleton is formed, although constant remodelling of the skeleton in response to changes in mechanical loading or bone fractures continues throughout life.

This process of bone modelling and remodelling is mediated by osteoblasts, osteoclasts, and osteocytes. Osteoblasts line the surface of the bone and are responsible for synthesising, secreting, and mineralising the bone matrix. Osteoblasts that become trapped within bone further differentiate into osteocytes which interact as a mechano-sensing network and direct the recruitment of osteoblasts and osteoclasts to local bone areas. Meanwhile, osteoclasts differentiate from hematopoietic precursors and migrate to areas of active bone remodelling to help degrade and resorb bone. The coordinated activity of these cells is essential to ensuring bone homeostasis as disturbances to this equilibrium can lead to disease (Table 1). Autophagy is one process that has been shown to be essential for maintaining this balance and for regulating bone and cartilage cell differentiation, formation, and function.

**Table 1:** Changes to autophagic proteins seen in specific skeletal disorders

Disease	Cell types involved	Phenotype	Autophagy proteins involved	Effect on autophagy	Ref
Paget’s disease of the bone	Osteoclasts	Disorganised and weakened bone; excessive bone resorption and accelerated bone turnover	SQSTM-1	Impaired autophagic flux	(Azzam et al. 2011; Ralston 2013)
Osteopetrosis	Osteoclasts	Increased bone mineral density; impaired bone resorption; reduced lysosomal trafficking and acidification	PLEKHM1	Altered autophagy activity; reduced MAP1LC3-II turnover	(Bo et al. 2016; Shen et al. 2018)
Osteogenesis imperfecta	Osteoblasts	Fragile and brittle bones; defective type I collagen production	ATG7, ATG5, BECN1, MAP1LC3-II, CTSK	Increased autophagy activity	(Besio et al. 2018; Gioia et al. 2012)
Osteoporosis	Osteoclasts, osteoblasts	Progressive loss of bone mass; increased bone fragility and fracture risk	ATG5, ATG7, ATG12, BECN1, PRKAA2, PIK3C3, IFNA13, GABARAPL1	Increased autophagy activity	(Zhang et al. 2010)
Glucocorticoid- induced osteoporosis	Osteoblasts and osteoclasts	Reduced bone density; increased fracture risk; reduced osteoblast proliferation; enhanced osteoclast survival	MAP1LC3	Increased autophagy activity	(Xia et al. 2010)
Osteoarthritis	Chondrocytes and osteoblasts	Progressive loss of articular cartilage; increased chondrocyte apoptosis; synovial inflammation; stiffening of joints	ULK1, BECN1, MAP1LC3	Decreased autophagy activity; impaired autophagy flux	(Carames et al. 2010; Cheng et al. 2017)
Multiple sulfatase deficiency	Chondrocytes	Skeletal dysplasia; deficiency in sulphate removal from GAGs	MAP1LC3	Increased autophagosomes due to defective fusion with lysosomes	(Lieberman et al. 2012)

### Autophagy in bone formation

Osteoblasts are the primary cell type responsible for bone formation and both their survival and function are regulated by autophagy. The differentiation of osteoblasts from MSCs is regulated by the transcription factors RUNX2 and SP7 (also known as Osterix). During this process, studies have shown that autophagy is upregulated to help these cells survive the hypoxic bone environment and to combat oxidative stress (Nollet et al. 2014), as manipulation of autophagic activity levels positively correlates with osteoblast survival (Li et al. 2017).

Beyond survival, autophagy activity is closely linked to osteoblast mineralisation. Osteoblasts form mineralised bone through the deposition of hydroxyapatite crystals into the collagen based bone matrix. As the matrix matures, these crystals are organised into a lattice structure within the collagen, forming bone. These hydroxyapatite crystals have been identified in autophagic vacuoles within osteoblasts, and the inhibition of autophagic flux has been shown to block the outward extrusion of these minerals (Nollet et al. 2014). Further to this, depletion or deletion of ATG5, ATG7 or Beclin1, which are essential for autophagosome formation, have all been shown to cause decreased bone mass and mineralisation (Nollet et al. 2014; Zahm et al. 2011). In the case of targeted ATG7 loss in mice, an increased number of bone fractures were recorded and suggested to be associated with induced ER stress and decreased osteoblast numbers (Li et al. 2018; Piemontese et al. 2016). Meanwhile, another in vitro study showed that deletion of FIP200 in osteoblasts impaired their terminal differentiation, inhibiting bone formation, and causing an osteopenia phenotype (Liu et al. 2013).

There are several bone disorders linked to osteoblast dysfunction whose pathogenesis has also been linked to autophagy dysregulation, such as Osteogenesis Imperfecta and osteoporosis (Table 1). For all bone disorders which are caused by excessive and disorganised or insufficient bone formation, treatment options are limited. This is because there are few drugs available which can effectively and safely target and promote osteoblast numbers and activity (Kawai et al. 2011; Riggs and Parfitt 2005). Therefore, influencing osteoblast activity through an alternative target, such as autophagy, could be a useful therapeutic mechanism, emphasising the need for more research into understanding the role of autophagy in osteoblast development and function.

### Autophagy in bone resorption

At sites of bone remodelling, hematopoietic mononuclear myeloid stem cells (HSCs), primarily residing in the bone marrow, can differentiate into osteoclasts and migrate to the bony tissue surface in a process mediated by colony-stimulating factor 1 (CSF-1) and RANK ligand (RANKL) (Zhao et al. 2007). Once differentiated and activated, osteoclasts attach to the bone surface, forming a seal via actin rings and begin bone resorption through the secretion of lysosomal proteases, metalloproteinases, and cathepsin K (CTSK). In mice, autophagy has been shown to play a role in osteoclast differentiation as HSC-specific loss of ATG7 caused increased genomic and cellular damage to HSCs and a failure to differentiate (Mortensen et al. 2011), indicating that autophagy may be important for the maintenance of HSCs. In addition to differentiation, autophagy helps osteoclasts survive in the locally hypoxic environment of the bone surface. In vitro studies have shown that in hypoxic environments, autophagy is upregulated in osteoclasts to reduce cell stress and to protect against apoptosis (Wang et al. 2011; Zhao et al. 2012). Correspondingly, increased autophagy activity enhances osteoclast differentiation (Shi et al. 2015).

Autophagy has also been shown to be involved in osteoclast activity and function. Osteoclasts resorb bone through the secretion of matrix-degrading molecules onto bone via secretory lysosomal vesicles. They have a characteristic ruffled border where the exocytosis of lysosomes occurs, and these lysosomes have been shown to be labelled with MAP1LC3 (DeSelm et al. 2011). Through the use of conditional knockout mouse models, roles for the ATG conjugation machinery in osteoclast formation and resorptive activity have been suggested (DeSelm et al. 2011). Loss of ATG5 and ATG7 has been shown to impair ruffled border formation and lysosomal trafficking and secretion, causing a reduction in bone-resorption capacity, and increasing trabecular bone volume. These mice also showed a decrease in MAP1LC3 and RAB7 (a RAB GTPase) localisation to the ruffled border and inhibited CTSK release, which likely explains the effect on resorptive activity. Similarly, MAP1LC3A – a specific sub-form of MAP1LC3 – was knocked down, both actin ring formation and CTSK release were blocked, thereby inhibiting resorption activity (Chung et al. 2012). Taken together, these data suggest that ATG proteins have a clear role in regulating osteoclast activity. This is further highlighted in the pathogenesis of bone disorders caused by defective osteoclast function which are also linked to mutations in autophagy related proteins, as mentioned in Table 1.

### Autophagy in bone maintenance

Osteocytes are terminally differentiated cells formed from osteoblasts which have become trapped within the bone matrix. They are vital for bone health and maintenance and are responsible for regulating the bone remodelling process. Through the extension of dendrite-like processes within the bone matrix, osteocytes connect to form a vast network which detects and responds to hormonal and mechanistic changes within the bone environment by directing the recruitment of osteoblasts and osteoclasts to local bone areas.

Just as autophagy has a key role in osteoblast differentiation and function, autophagy has been shown to be important for osteocyte health and maintenance. Firstly, during the osteoblast to osteocyte transition, the cells must undergo a dramatic change in cell shape and composition which requires an active recycling of organelles (Dallas and Bonewald 2010). Secondly, a study using human and rat bone tissue demonstrated that osteocytes show an accumulation of MAP1LC3 puncta and that this expression is higher in osteocytes than osteoblasts (Zahm et al. 2011). This indicates a high basal level of autophagy which is likely to be necessary to survive the nutrient and oxygen poor environment of the bone matrix.

Looking into the role of autophagy in osteocyte functioning, when autophagy activity was inhibited in mice by the osteocyte specific deletion of ATG7, there was a significant decrease in bone mass which was associated with reduced osteoblast and osteoclast numbers, and a disturbance in bone homeostasis (Onal et al. 2013). Similarly, in mice with ATG7 deficient osteoblasts, osteocytes showed decreased cellular projections and reduced ER degradation and turnover (Piemontese et al. 2016). Together, these results indicate a clear role for autophagy in osteocyte function, whilst also demonstrating the level of interaction between these skeletal cell types and how the dysfunction of one can impact the activity of others. This, therefore, has a significant impact upon bone health and homeostasis and should be an important consideration when studying bone disorders and selecting possible drug targets.

### Autophagy in cartilage formation and maintenance

Alongside bone cells, cartilage forming chondrocytes also play a critical role within skeletal development and function. Chondrocytes are responsible for forming both the initial cartilaginous skeleton during endochondral ossification and the articular cartilage layer between bones, which enables fluid joint movement. As with the other skeletal cell populations, autophagy has been shown to be a vital process for chondrocyte differentiation, function and survival (Vuppalapati et al. 2015; Zhang et al. 2013).

During endochondral bone formation, chondrocytes form the cartilage anlage of the future bone through the secretion of a collagen-rich matrix. This process continues until the chondrocytes reach a non-proliferative, hypertrophic state at which point the cells undergo apoptosis, triggering the resorption of cartilage and its mineralisation into bone by invading osteoblasts (Berendsen and Olsen 2015). Some chondrocytes remain within regions near to the end of the forming bone known as growth plates, where the chondrocytes continue to proliferate and secrete a cartilage matrix to enable longitudinal bone growth via ossification. During this process of chondrocyte proliferation and differentiation, in vitro studies have shown there is a positive correlation with levels of autophagy activity (Vuppalapati et al. 2015), and maturing chondrocytes show high MAP1LC3 expression (Srinivas et al. 2009). In growth plate chondrocytes, mice with a chondrocyte specific deletion of ATG7 showed impaired matrix secretion due to the retention of synthesised type II procollagen (a major component of cartilage matrix) within the ER (Cinque et al. 2015). Meanwhile another study showed that the conditional loss of ATG5 or ATG7 in mice enhanced chondrocyte cell death and decreased cell proliferation resulting in reduced growth plate activity and growth retardation (Vuppalapati et al. 2015).

Joint articular cartilage is retained throughout life, although due to the limited regenerative and repair capabilities of articular cartilage, homeostatic mechanisms such as autophagy are vital for its maintenance and preservation (Barranco 2015; Zhang et al. 2013). For example, healthy human cartilage shows a high expression of key autophagy factors such as ULK1, Beclin1, and autophagosome-associated MAP1LC3-II, indicating that autophagy is a constitutively active mechanism within cartilage (Caramés et al. 2010). Meanwhile, mice with a conditional knockout of ATG5 in chondrocytes showed increased articular chondrocyte cell death, which escalated with age, and an accumulation of p62 in the articular cartilage, indicating abrogated autophagic flux (Bouderlique et al. 2016). By 1 year of age, these mice had experienced significant or complete loss of articular cartilage at joint sites and severe development of the joint disease osteoarthritis (OA). Together, these results suggest that autophagy may play a protective role against cartilage degradation by maintaining chondrocyte health and survival. This is further supported by data demonstrating that patients with osteoarthritis, which is characterised by the progressive loss of articular cartilage, show decreased expression of key autophagy markers which continue to decline further as the severity of the disease increases (Carames et al. 2010).

## Zebrafish as a model to study autophagy during skeletal development and pathology

As discussed above, it is clear that autophagy is an important player within bone and cartilage cell development and maintenance, and that its activity is vital for sustaining skeletal homeostasis. Indeed, there are multiple skeletal disorders that are triggered by an imbalance in bone or cartilage cell activity with accompanying dysregulated autophagy activity. Many of these disorders are chronic and debilitating, and currently have limited treatment options available. Therefore, expanding our understanding of the cellular and molecular processes that are central to the co-ordination of bone and joint development, such as autophagy, will be crucial for the advancement of new therapeutics for these diseases, as well as for broadening our understanding of their pathogenesis.

Much has been learnt about the skeletal system and its associated disorders through the use of animal models (Gomes and Fernandes 2011). In vivo models offer obvious advantages over in vitro cell models for skeletal research as the complex, moveable, three-dimensional structure of bones and joints cannot be fully recapitulated within an in vitro system. Equally, the effects of other cell and tissues types and their related secretions on cartilage and bone cells cannot be recaptured within a unicellular system. Whilst a number of different animals have been used as models for bone research, mouse models remain the most extensively used due to their generally lower husbandry cost, fast generation times, ease of handling and genetic tractability (Sommer et al. 2019). However, despite these advantages, rodent models do have some inherent limitations for bone and autophagy-based research such as, differential bone loading compared to humans, and a lack of visual accessibility at a cellular level and during early developmental stages.

Increasingly, zebrafish (*Danio rerio*) are being recognised as a useful alternative to rodent models for skeletal and autophagy research. Firstly, zebrafish are highly fecund, with a single pair able to lay up to 300 eggs a week, which develop externally as optically translucent larvae. This allows for the study of both cellular and gross morphological changes during early development, without the need for invasive experimental techniques or animal sacrifice. Secondly, zebrafish are highly genetically tractable, as through the use of genetic tools such as TALEN (Bedell et al. 2012) and CRISPR/Cas9 (Talbot and Amacher 2014) embryos can be injected with constructs at the single cell stage to generate transgenic or genetically altered zebrafish lines. As these tools continue to improve, and with access to a fully sequenced genome, it is possible in zebrafish to efficiently and specifically target multiple genes in a high-throughput manner (Liu et al. 2019). It is through such methods that many knockout and reporter lines have been developed and used to model specific diseases or to visualise and track specific proteins or cell types, such as bone cells or autophagy related proteins.

As vertebrates, zebrafish show a high genetic similarity to humans (Kabashi et al. 2011), and all of the core mammalian autophagy-related proteins can be found within the zebrafish genome, with the overall amino acid identity between these and their human counterparts ranging between 40 and 96% (Mathai et al. 2017). This high degree of conservation indicates that the autophagy pathway operates in a very similar way within zebrafish compared to humans and has enabled the development of many mutant and transgenic autophagy zebrafish lines (Table 2).

**Table 2:** List of transgenic and mutant zebrafish lines that can be used to study autophagy

Gene	Protein description	Effect on autophagy	Line name	Ref
*Map1lc3b*	Marker for autophagosomes	Reporter – Enables visualisation of autophagosomal structures; under high magnification can be seen as distinct puncta	*Tg(CMV:EGFP-map1lc3b)*	(He et al. 2009)
*Gabarap*	Marker for autophagosomes	Reporter – A functional homologue of Map1lc3; enables visualisation of autophagosomal structures, under high magnification can be seen as distinct puncta	*Tg(CMV:EGFP-gabarap)*	(He et al. 2009)
*Map1lc3b*	Marker for autophagosomes	Reporter – Tandem fluorescent tag allows for monitoring of autophagic flux and acidity of autolysosomes	*Tg(CMV:EGFP-map1lc3b; CMV:mCherry-map1lc3b)*	(Sasaki et al. 2014)
*Gabarap*	Marker for autophagosomes	Reporter – Tandem fluorescent tag allows for monitoring of autophagic flux and acidity of autolysosomes	*Tg(CMV:EGFP-gabarapa; CMV:mCherry-map1lc3b)*	(Sasaki et al. 2014)
*Sqstm1*	Autophagy receptor	Reporter—Enables visualisation of autophagosomal structures	*Tg(pT2-mCherry-sqstm1)*	(Sasaki et al. 2017)
*Lamp1*	Lysosomal membrane marker	Reporter – Enables visualisation of lysosomes	*Tg(pT2-lamp1-mCherry)*	(Sasaki et al. 2017)
*Atg7*	Processes Map1Lc3 ready for conjugation	Mutant – Full protein KO; shows larvae lethality; reduction in Map1Lc3-II and accumulation in p62	*atg7*^*sa14768*^	(Mawed et al. 2019; Siddiqi et al. 2019)
*Atg12*	Conjugates Map1Lc3 to PE	Mutant – Full protein KO; shows increased p62 accumulation indicative of autophagy inhibition	*atg12*^*ecn3*^	(Lu et al. 2019)
*Beclin1*	Involved in autophagy initiation	Mutant – Full protein KO; shows larvae lethality; reduction in Map1Lc3-II and accumulation in p62	*beclin1Δ8*	(Dong et al. 2020; Mawed et al. 2019)
*Dram1*	Localises to lysosomes and promotes autophagic flux	Mutant – Full protein KO; shows accumulation of Map1Lc3 and p62 under induced autophagy; reduced targeting of bacteria to lysosomes	*dram1*^*ibl53*^	(Zhang et al. 2020)
*Epg5*	Rab7a effector	Mutant – Full protein KO; shows impaired autophagic flux due to defective degradation of autolysosomes	*epg5*^*ia31*^	(Meneghetti et al. 2019)
*Lamp2*	Lysosomal membrane marker	Mutant – Full protein KO; shows impaired autophagic flux due to disrupted autophagosomal fusion with lysosomes	*lamp2*^*e2*^	(Dvornikov et al. 2019)
*Optineurin*	Autophagy receptor	Mutant – Full protein KO; shows decreased levels of Map1Lc3-II and p62	*optn*^*ibl51*^	(Zhang et al. 2019)
*Spns1*	Lysosomal H^+^ transporter	Mutant – Full protein KO; shows accumulation of Map1Lc3-I and II; impaired autophagic flux due to improper autolysosomal degradation	*spns1*^*hi891*^	(Sasaki et al. 2017)
*Sqstm1*	Autophagy receptor	Mutant – Full protein KO; shows decreased levels of Map1Lc3-II and optineurin	*p62*^*ibl52*^	(Zhang et al. 2019)

Despite clear structural differences, zebrafish also share similar skeletal physiology to mammals, including the same joint types and joint components such as joint cavities, articular cartilage and synovial membranes (Askary et al. 2016). This has been most widely shown in the larval zebrafish jaw which has been extensively studied and remains one of the main joint sites used to model joint development. Additionally, the overall molecular mechanisms underlining vertebrate skeletal segmentation, joint development, and fin/limb development are very similar and are likely to have been conserved across species (Crotwell and Mabee 2007).

Many human skeletal disorders can be modelled in zebrafish and can recapitulate the phenotypes seen in higher vertebrates. For example, equivalent models for disorders such as osteogenesis imperfecta, scoliosis, osteoporosis, Stickler syndrome, and osteoarthritis are available in zebrafish (Askary et al. 2016; Carnovali et al. 2019; Lawrence et al. 2018; Mackay et al. 2013). Additionally, as zebrafish develop osteoarthritis naturally during ageing, the pathogenesis of the disease and its common symptoms such as increased spinal deformities, vertebral dislocations, and fractures, and the formation of osteophytes can be easily explored (Hayes et al. 2013). Taken together, these data demonstrate that zebrafish are representative, relevant, and useful models for the study of bone and joint development, the pathology of skeletal disorders, and the genes involved in either processes. They also present some unique advantages for the dynamic and real-time study of autophagy activity and expression within different skeletal cell types and systems from early development to adulthood.

## Current tools available to study autophagy in zebrafish

### Transgenic and mutant zebrafish lines

Given the genetic and physiological similarities between humans and zebrafish, multiple transgenic and mutant lines targeting key autophagy and skeletal genes have been developed in zebrafish (for common skeletal zebrafish lines, see (Bergen et al. 2019)). Although these lines have been well used for studies within their respective fields, much less research has been done using autophagy and skeletal zebrafish lines in combination.

**Fig. 3:**
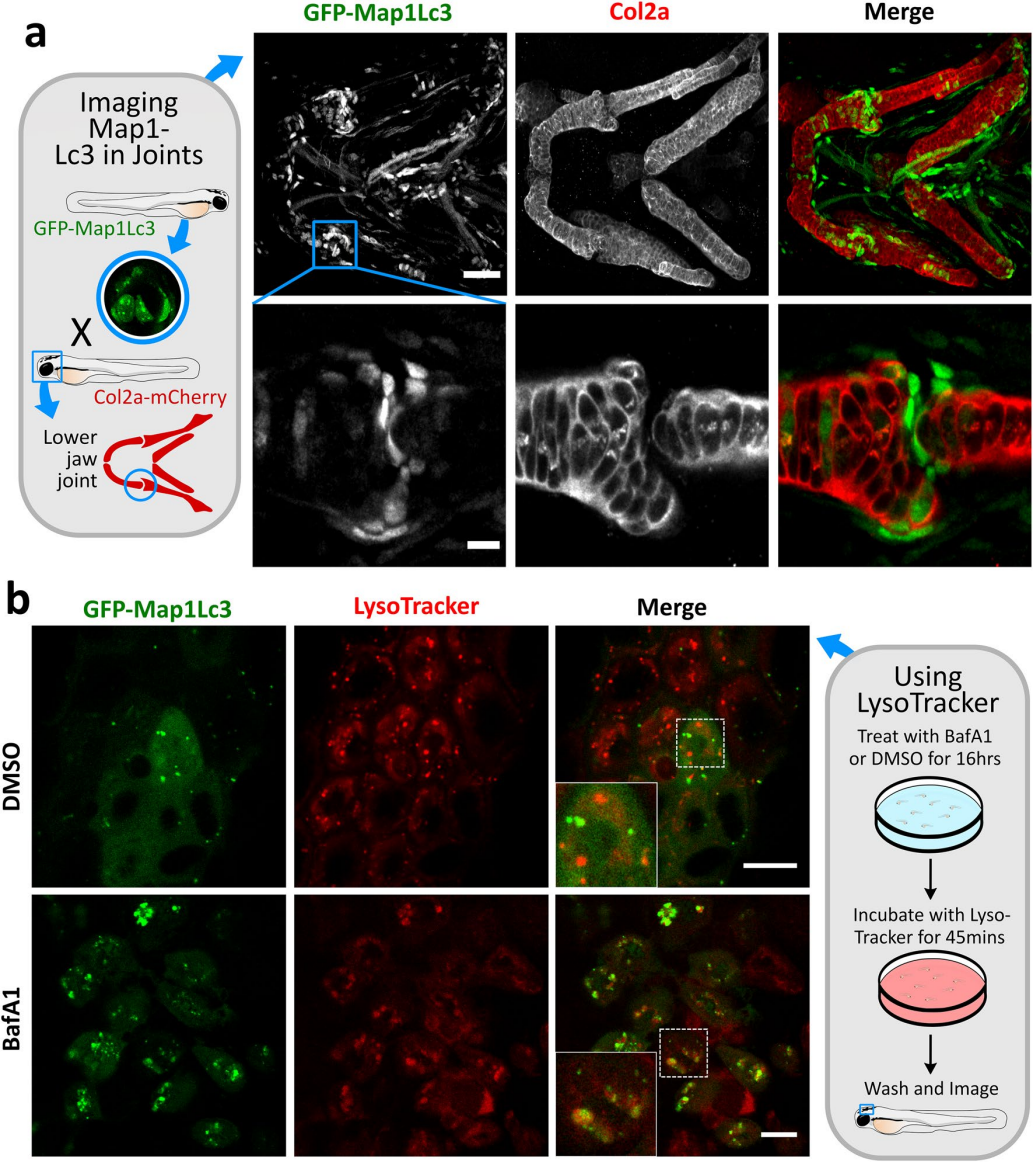
Examples of how GFP-Map1Lc3 transgenic zebrafish line can be used to study autophagy in a skeletal context, **a** Confocal images of the lower jaw and lower jaw joint of a transgenic *CMV:EGFP-map1lc3b* zebrafish at 3 days post fertilisation (dpf), immunostained for collagen Type II (red) and anti-GFP for lc3 (green). Scale bar = 50 µm and 10 µm, respectively. **b** Confocal images of epidermal cells in transgenic *CMV:EGFP-map1lc3b* zebrafish imaged at 5dpf after treated with 100 µl BafilomycinA1 or DMSO for 16 h followed by 45 min live staining in red LysoTracker dye. Inset boxes show zoom of white checked box. Scale bar = 10 µm

The first transgenic autophagy reporters generated in zebrafish were the GFP-Map1Lc3 and GFP-Gabarap transgenic lines, with reporters expressed under the control of the constitutive cytomegalovirus (CMV) promoter (He et al. 2009) (Table 2). Both Map1Lc3 and Gabarap are homologues of yeast Atg8, and each form a subfamily of proteins in mammals and fish. In mammalian cells, the MAP1LC3 and GABARAP family members act cooperatively to enable autophagosome formation and/or cargo recognition, and as such, are equally useful for measuring autophagy in vivo and in vitro. However, overall, Map1Lc3 is the most widely used marker for identifying and visualising autophagy activity. During autophagy, GFP-Map1Lc3 acts like endogenous Map1Lc3 and becomes conjugated to PE in the developing phagophore and remains associated with the autophagosome until its full closure (Fig. 1). Using fluorescence microscopy, this lipidated form of GFP-Map1Lc3-II can be visualised as puncta or as ring-like structures as shown in Fig. 3 (Kabeya et al. 2000; Mizushima et al. 2004).

The GFP-Map1Lc3 transgenic zebrafish line has been used in several studies to explore the role of autophagy in bacterial clearance (Mostowy et al. 2013), blastema formation following fin amputation (Varga et al. 2014), and in liver homeostasis (Cui et al. 2012). With regards to skeletal biology, this line can also be used to explore the expression pattern of autophagy during early development and to identify cell types showing high expression of autophagy activity. As shown in Fig. 3, we were able to identify that during development, zebrafish show high expression of GFP-Map1Lc3 around joint sites, and that the cells in the joint interzone specifically show increased GFP-Map1Lc3 expression compared to the surrounding cells. Given the optical clarity of zebrafish, these fish can be imaged live under anaesthetic, and the expression of Map1lc3 can be tracked throughout development in the same fish.

Moreover, through the use of skeletal cell specific transgenic lines, the expression of GFP-Map1Lc3 can be correlated to a specific cell type. For example, transgenic lines expressing Col10a1 or sp7 can be used to label osteoblasts at different stages of differentiation; transgenic GFP-trap fish can be used to label osteoclasts; whilst fluorescent labelling of type II collagen can be used to monitor chondrocytes and cartilage development and formation. Similarly, there are many antibodies and transgenics available for the labelling of muscles, collagens, tendons, and ligaments, enabling the expression of Map1Lc3 to be explored in many different cell types related to the skeletal system. This can allow for the identification of patterns in autophagy activity during skeletal patterning and differentiation, either in normal development, in response to the mutation of genes of interest, or during pharmacological (Seda et al. 2019) or mechanical manipulation (Brunt et al. 2016). These data could be correlated to phenotypes seen in disease models to explore how autophagy activity may be being altered within different bone disorders. There are also several autophagy-specific knockout zebrafish lines which have been shown to block or impair autophagy activity (Table 2). These enable investigations of how loss of autophagy activity affects the differentiation and function of skeletal cells and the gross development and performance of the skeleton.

While expression of GFP-Map1Lc3 can be very useful for identifying where autophagy may be upregulated, its expression alone cannot be used to determine autophagic flux or dynamics. For example, an increase in Map1Lc3 puncta could be due to elevated autophagy activity, and/or due to impaired lysosomal clearance (i.e. impaired autophagic flux) (Klionsky et al. 2016). To measure autophagic flux in zebrafish, lysosomes can be labelled with either a dye such as LysoTracker (Fig. 3b), or a fluorescent probe, such as mCherry-Lamp1 or Lamp2 (Sasaki et al. 2017). In this way, colocalisation between GFP-Map1Lc3 and a lysosomal marker can be used to assess the level of autophagic activity. Similarly, drugs which block autolysosome formation (e.g. lysosomal H^+^-ATPase inhibitors), and therefore prevent the turnover of Map1Lc3 puncta, can also be applied to assess autophagic flux (Fig. 1). However, when using autophagy modulators it is important to consider their full effects in vivo*,* as several target other non-autophagic pathways such as the endocytic pathway, or cause other indirect effects (Klionsky et al. 2016).

Dual fluorescent probes, or tandem-tags, such as mCherry-GFP-Map1Lc3, are also very useful for analysing autophagy flux. Unlike the fluorescent GFP probe, mCherry is less sensitive to the acidic environment of the lysosome, and therefore is not quenched. By measuring the proportion of yellow (green and red puncta together) and red puncta (autophagosomes and autolysosomes, respectively), the level of autophagic flux activity can be estimated. Together, these tools provide a more accurate way to measure autophagy activity and differences in autophagy levels quantitatively between different fish. Indeed, these tools have been used in zebrafish studies to determine how autophagic flux is affected by specific genetic mutations within bone and cartilage cells (Hu et al. 2019; Santos-Ledo et al. 2017). These studies showed that changes to bone and cartilage cell differentiation and functioning were in part due to dysregulated autophagy activity. Therefore, these tools could also be used to help establish whether bone disorders alter autophagic flux within specific cells and to explore the potential benefit of autophagy-modulating drugs at addressing this imbalance.

Recently, there has been increased interest in using zebrafish scales as model for imaging bone cell dynamics (Bergen et al. 2019; Pasqualetti et al. 2012). Although their structure is simpler compared to mammalian bones, several studies have shown that osteoclasts and osteoblasts can respond to hormones and other substances indicating that their activity and fundamental regulation is unchanged (Carnovali et al. 2016; de Vrieze et al. 2015; Park et al. 2016). Therefore, zebrafish scales offer a new in vivo bone model for the live imaging of osteoclasts and osteoblasts in fluorescent transgenic lines. Looking ahead, this model could be used to study autophagy dynamics in bone cells using transgenic autophagy lines, or to screen autophagy modulating drugs for their effects on bone cell activity.

### High-throughput drug screening

Over the last 20 years, zebrafish larvae have become an established model for high throughput chemical screens (Cully 2019; Rennekamp and Peterson 2015). Utilising the transparency, small size, and ease of drug administration, zebrafish can provide both the high-throughput capabilities of an in vitro system with the full complexities of whole organism biology. Combining this system with high-resolution imaging and fluorescent reporters/dyes means that the effects of putative drugs on a specific cellular pathway or disease mutation can be rapidly quantified, assessed and validated (Early et al. 2018; Mathias et al. 2012; Walker et al. 2012). To date, eleven compounds have been identified through zebrafish screens as potential therapeutics, of which nine are in or about to enter early clinical trials (Cully 2019), including one for the connective tissue disorder, Fibrodysplasia ossificans progressive. As several skeletal disorders can be accurately modelled in zebrafish, this system could be applied as a primary screening platform for identifying and testing potential therapeutics for these disorders. Using autophagy reporter lines, it could also be used to identify new autophagy modulating drugs or to test the efficacy of current modulators on skeletal disease pathology. For example, the GFP-Map1Lc3 line has been used to validate the effect of potential autophagy enhancers, such as AUTEN-67, identified through small-molecule library screens (Papp et al. 2016). Meanwhile, Khuansuwan et al. used a neuron-specific GFP-Map1Lc3b line to validate the effect of two, in-trial drugs for Parkinson’s as autophagy modulators (Khuansuwan et al. 2019).

As mentioned previously, interest is growing in the use of zebrafish scales as an ex vivo model. This model has the advantage that screens can be performed on scales harvested from a single fish thereby reducing intra-individual variation, and it allows for compounds to be tested in the context of homeostasis within a mature tissue (de Vrieze et al. 2015). Already, this model has been used to study the effect of different drugs on bone cells (Park et al. 2016), and to screen compound libraries to identify new osteo-anabolic and catabolic drugs (de Vrieze et al. 2015). Similarly, this system has been proposed for the identification of drugs for osteoporosis, whereby scales collected from fish with osteoporosis-specific mutations are used to screen for possible therapeutic drugs (Bergen et al. 2019). Together, both of these systems show enormous potential as primary testing platforms and demonstrate how zebrafish can be used to provide fast, functional validation of prospective new drugs.

### Bone repair and regeneration assays

**Fig. 4:**
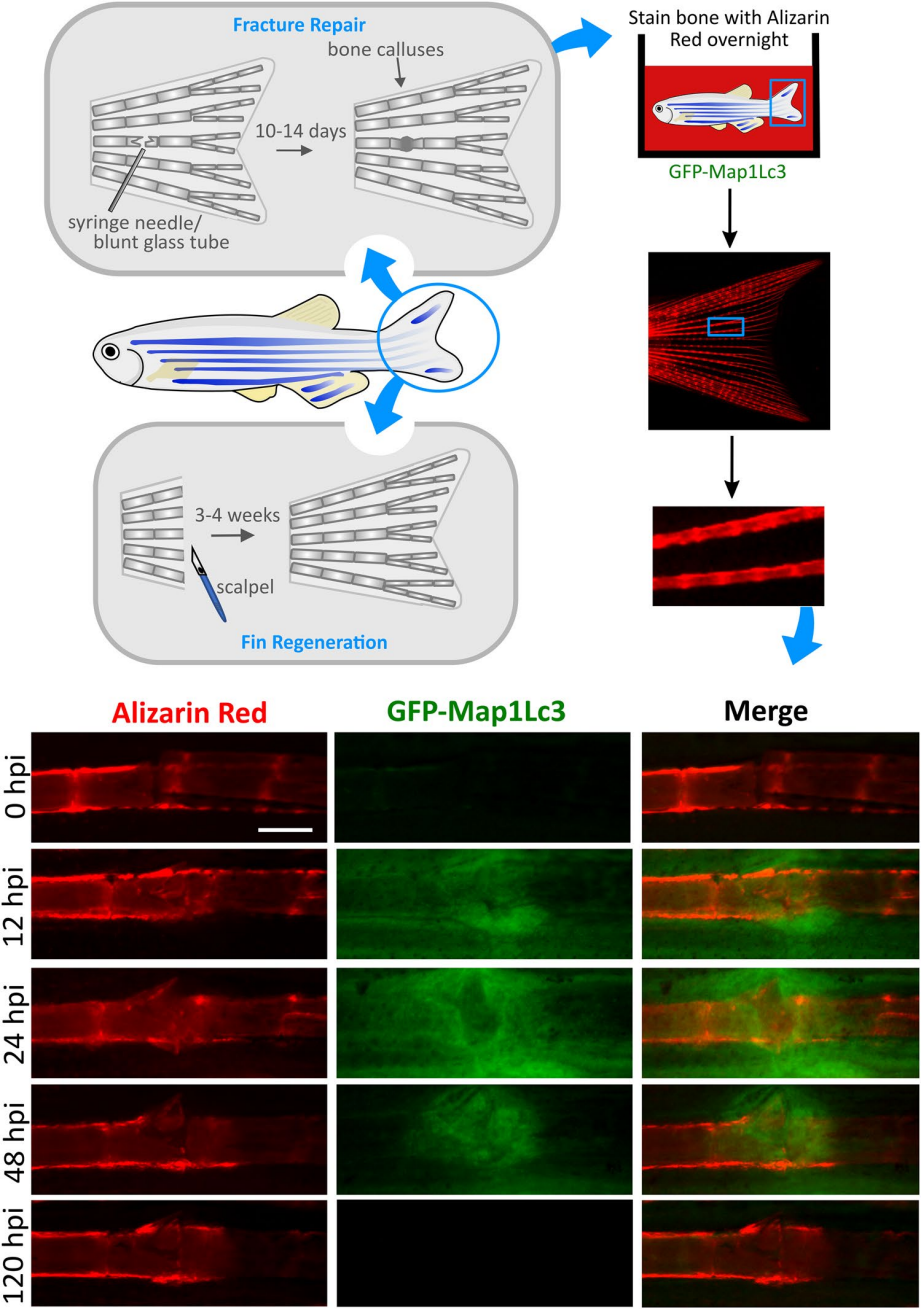
Using GFP-Map1Lc3 transgenic zebrafish line to study the role of autophagy in fin fracture repair and bone regeneration – *Top,* Schematics depicting how bone fracture repair and regeneration assays can be performed in zebrafish and how live staining of bone can be performed in adult zebrafish using Alizarin Red. *Bottom,* Fluorescent stereomicroscope images of a fracture repair assay performed in a transgenic *CMV:EGFP-map1lc3b* zebrafish at 6 months post fertilisation, live stained with Alizarin Red (red). *Dpi* days post injury; scale bar = 200 µm. Figure made in collaboration with Miss Lucy McGowan

As zebrafish fins and scales remain optically accessible throughout adulthood, zebrafish provide a useful tool for dynamically observing the factors involved in bone regeneration and bone repair (Fig. 4). Similar to other teleosts, zebrafish can regenerate parts of their body following amputation. In the fins, this regeneration process is relatively quick and within two weeks all major tissue, including bone, joints, and nerves are largely restored (Watson and Kwon 2015). Using a fin regeneration assay, Varga et al*.* explored the role of autophagy within this process and showed that the genetic and pharmacological inhibition of autophagy impairs fin regeneration (Varga et al. 2014). This highlighted an important role for autophagy in tissue patterning and renewal. Further investigations into this process could be helpful for identifying how manipulation of autophagy can be used to promote bone and cartilage cell renewal and replacement, especially during ageing.

Zebrafish are also a useful model for fracture repair studies as they show a fracture healing response with callus formation which is very similar to mammals (Sousa et al. 2012; Tomecka et al. 2019). Using the GFP-Map1Lc3 line and the live bone stain, Alizarin red, we were able to perform a fin fracture assay which showed that the expression of Map1Lc3 increases during the early stages of fracture repair (Fig. 4). This indicates that the autophagy activity may be upregulated during the repair response and suggests a role for autophagy in bone repair. Similarly, these tools could also be utilised within the zebrafish scale system which was recently used to model the fracture repair response (Kobayashi-Sun et al. 2020). Using both of these models, the impact of autophagy modulating drugs, such as rapamycin, on the rate of fracture repair could be explored. Given that a number of bone disorders result in the development of weakened and fracture-prone bones, this could be particularly useful model for discovering potential bone repair therapies.

## Future directions and conclusion

Existing studies have established zebrafish as a powerful model for studying vertebrate development and modelling genetic diseases, including various skeletal disorders (Carnovali et al. 2019; Kwon et al. 2019). Use of zebrafish in autophagy studies is more recent but growing nonetheless, and draws on the ease and plasticity of live imaging options they present, which simply cannot be paralleled in the more commonly used rodent autophagy models. As outlined above, initial studies using zebrafish autophagy models have already provided a new insight into the role of autophagy in skeletal cell differentiation and functioning, bone repair and regeneration, and drug discovery.

As these tools are further refined, the advantages of this model can be further exploited for autophagy research. For example, the development of conditional knockout lines of autophagy related genes for skeletal cells would enable the role of autophagy in these cells to be explored more accurately. Moreover, the development of inducible knockout lines would be especially helpful as the loss of essential autophagy proteins has proved to be developmentally lethal in several models (Dong et al. 2020; Mawed et al. 2019).

The use of cultured zebrafish scales as an ex vivo model is a very recent development that shows great potential for exploring the dynamics between autophagy and bone cell activity and function. As a flat but 3D whole skeletal preparation, autophagy assays similar to those done within in vitro models could be performed in zebrafish scales. This would help deepen our understanding of autophagy activity within osteoblasts and osteoclasts, and how different drugs or genetic mutations impact upon autophagy flux. The potential use of cultured scales in high throughput drug screening is also still yet to be fully explored. Together with zebrafish larvae, the scale system could help bridge the gap between modelling the complex biology of bones and rapidly testing and validating potential drugs.

As the tools available for genetic manipulation and in vivo imaging in zebrafish continue to improve, this model will be fundamental to progressing our understanding of autophagy cell biology and the significance of its role in skeletal development and pathology. Whilst it should be appreciated that not all observations from zebrafish, as with any animal model, are translatable to the human condition, zebrafish can still make a valuable contribution towards understanding the interactions between the autophagy pathway and the skeletal system, and in developing new therapeutics.
